# The Importance of the Built Environment in Person-Centred Rehabilitation at Home: Study Protocol

**DOI:** 10.3390/ijerph16132409

**Published:** 2019-07-06

**Authors:** Maya Kylén, Lena Von Koch, Hélène Pessah-Rasmussen, Elizabeth Marcheschi, Charlotte Ytterberg, Ann Heylighen, Marie Elf

**Affiliations:** 1 School of Education, Health and Social Studies, Dalarna University, SE-791 88 Falun, Sweden; 2 Karolinska Institutet, Department of Neurobiology, Care Sciences and Society, SE-141 83 Huddinge, Sweden; 3 Karolinska University Hospital, SE-141 86 Stockholm, Sweden; 4 Skåne University Hospital, Department of Neurology and Rehabilitation Medicine, 221 85 Lund, Sweden; 5 Lund University, Department of Clinical Sciences Lund, Neurology, 221 84 Lund, Sweden; 6 Arkitektur och samhällsbyggnadsteknik, Byggnadsdesign, ACE, Chalmers University, SE-412 96 Gothenburg, Sweden; 7 KU Leuven, Department of Architecture, Research[x]Design, 3001 Leuven, Belgium

**Keywords:** rehabilitation, person-centred care, person–environment fit, mixed-methods design, housing

## Abstract

Health services will change dramatically as the prevalence of home healthcare increases. Only technologically advanced acute care will be performed in hospitals. This—along with the increased healthcare needs of people with long-term conditions such as stroke and the rising demand for services to be more person-centred—will place pressure on healthcare to consider quality across the continuum of care. Research indicates that planned discharge tailored to individual needs can reduce adverse events and promote competence in self-management. However, the environmental factors that may play a role in a patient’s recovery process remain unexplored. This paper presents a protocol with the purpose to explore factors in the built environment that can facilitate/hinder a person-centred rehabilitation process in the home. The project uses a convergent parallel mixed-methods design, with ICF (International Classification of Functioning, Disability and Health) and person–environment theories as conceptual frameworks. Data will be collected during home visits 3 months after stroke onset. Medical records, questionnaires, interviews and observations will be used. Workshops will be held to identify what experts and users (patients, significant others, staff) consider important in the built environment. Data will be used to synthesise the contexts, mechanisms and outcomes that are important to support the rehabilitation process at home.

## 1. Introduction

As a result of recent health-policy changes, the home is rapidly growing as the place for healthcare and is central to reforms in all OECD (The Organisation for Economic Co-operation and Development) countries and beyond [1,2]. Although the immediate surroundings (i.e., home and neighbourhood) are suggested as being key factors in the next generation of health reforms for creating person-centred care to promote autonomy, authority and choices [3,4], little is known about the facilitators and barriers for a person-centred rehabilitation process at home [5]. This topic requires attention because today, hospital stays are short; only acute care is delivered in high-technology units, and rehabilitation is usually performed outside the hospital setting [6]. Involving the patient and his/her significant others in a collaborative decision-making process is an essential element in today’s healthcare systems. In this process, it is important to consider not only the objectives of care but also the meanings of the place where care and rehabilitation activities take place [7]. The new healthcare reform assumes that persons prefer that care and rehabilitation takes place at home, but this assumption neglects the diversity of personal needs and the different meanings people attach to their homes, which may have an impact on functional recovery as well as on social, emotional and physical aspects of daily life [8]. To further develop evidence-based intermediate care models (i.e., alternatives to hospital care) and incorporate person–environment dynamics, research to understand how environmental factors relate to everyday life and recovering at home is crucial, especially for persons with long-term conditions such as stroke.

Stroke is a common disease and a leading cause of lifelong disability [9]. Persons with stroke are often from older age groups and require long-term healthcare [10]. Studies have shown that 80% of persons with stroke experience an adverse event the first year after discharge from inpatient rehabilitation [11]. The physical, cognitive and psychological impairments resulting from stroke can lead to a wide range of activity limitations and participation restrictions [12,13]. Stroke survivors and their families often report that the post-discharge period is stressful and challenging as they adjust to a new situation and new roles [12]. Returning to previous activities and ways of living is critical for well-being and is often considered a goal for rehabilitation [14]. The WHO stresses that the transition from inpatient care to home care should be person-centred [3] and that each dimension of a person’s health and well-being should be regarded as an interaction between the health conditions of the person and related environmental and personal factors. Although environmental psychology research suggests that human–environment interactions cannot be fully understood without considering the physical scenario in which rehabilitation activities are performed [15], the physical factors of the environment have largely been overlooked [5].

The built environment is essential for achieving high-quality healthcare. Evidence-based architecture is a relatively new field that aims to inform healthcare design through the best available scientific knowledge, i.e., the user’s requirements and experiences [16]. Research has shown that the built environment influences organizational factors, defines the context in which care processes and social interactions take place, and influences outcomes such as patients’ well-being and health [17]. Environmental factors such as views and colour schemes have been linked to more rapid recovery, less use of pain-relieving drugs and fewer sleeping disturbances. Related research has predominantly been performed in hyper-acute settings (Intensive care units, surgery) [18], and some attention has been devoted to specific groups such as older people in residential care units [19] or mental health environments [20]. However, regarding environments for stroke rehabilitation, research is scarce. We know very little about how the home setting and its design can promote or hinder a person’s health and well-being and optimize the health service processes.

Evidence suggests that the perspectives of persons with stroke regarding their service needs are not aligned with those of health professionals [21], even though Swedish healthcare states that services should be patient-centred and involve patients as active participants [22]. In addition, several studies have shown that the coordination and communication among healthcare providers is lacking, which may lead to preventable hospital readmissions, reduced quality of life, reduced patient safety and patient satisfaction (e.g., falls, medication errors), higher healthcare costs and an increased burden on significant others [23,24]. Persons with stroke represents a vulnerable population and overall, the literature suggests that there is a need for research to enhance the understanding for how to organize care and on how to design houses to support patients receiving such health services. Moreover, taken that the medical record system in Sweden is not designed to incorporate patient views [25], which aggravates possibilities for person-centred care, more research in this domain is urgently needed. The low user involvement in healthcare calls for the greater inclusion of the perspectives of experts and users [26], i.e., patients, significant others, staff, and interdisciplinary teams, in the development of new person-centred healthcare services, of which the built environment is an important part.

This protocol presents a project that will increase the knowledge on patients’ situation, including how the built environment can facilitate/hinder the recovery, person-centred care and rehabilitation in their home. The outcomes will be crucial for the enhancement of improved home health services for persons with stroke.

### 1.1. Aims

Our research protocol attends to the gap in knowledge about whether and to what extent features in the built environment can facilitate and/or hinder a person-centred rehabilitation process. The project aims to explore environmental factors in patients’ home settings and neighbourhoods and the relationship between these factors and patient outcomes. The specific aims are as follows:

1. To explore how environmental factors inside and outside the home can facilitate and/or hinder a person-centred rehabilitation process regarding roles and participation in meaningful activities.
2. To identify and describe how environmental factors in the home environment support stroke patients’ perceived health, self-efficacy and safety.
3. To explore whether and how stroke patients’ perceptions of home as a significant place interrelates with their rehabilitation process.
4. To explore whether and how the built environment has been taken into account during rehabilitation planning and to identify key success factors related to cross-site communication and collaboration between inpatient and primary care providers.
5. To explore how the built environment for stroke rehabilitation could be designed to support a person-centred rehabilitation process from the stroke unit to the home environment.

To explore these aims, we will be collecting data on environmental barriers/facilitators, cross-site communication and health outcomes of persons with stroke, significant others as well as healthcare professionals in Sweden. Collected qualitative and quantitative data will be used to synthesise the contexts, mechanisms and outcomes that are important to support a person-centred rehabilitation process in the context of the home. In doing so, this research will generate a framework needed to support a person-centred rehabilitation process and improve health and well-being for this vulnerable population.

### 1.2. Underpinning Theories and Concepts

The WHO’s ICF framework [27] underpins the project. The WHO underscores the significance of the environment for people’s health, which is viewed as an outcome of the complex dynamic interaction between functioning, i.e., bodily functioning/activity/participation, and contextual factors, i.e., personal and environmental factors.

In addition, the project adopts a holistic view of the environment, and it views the built environment, social environment and geographic location as important factors for a person-centred rehabilitation process [15]. We view the concept of home as something different from that of housing. A house is purely a space and a building, while a home is a place filled with personal experiences, meaning and social relationships, that over time transform space (house) into place (home) [28]. Such bonds are closely related to well-being and identity, and this is especially true for persons facing functional decline as the home can serve to preserve independence in everyday life [28]. A large number of concepts have been suggested to mirror these complex person–environment dynamics, among these concepts are place attachment and the meaning of home. Place attachment is defined as an emotional bond between an individual and a place, which supports the development of feelings such as sense of safety, self-efficacy, belonging and connectedness [29]. The concept meaning of home builds on place attachment theory and refers to symbolic representations of space and place and personal meanings related to one’s home. That is, the home represents individual meanings related to the individual’s personality and experience and is not only considered to fulfil objective functions [30]. In line with examining person–environment dynamics and adopting a person-centred approach, the project investigates place attachment and the meaning of home as potential factors for health outcomes among patients with stroke [31,32,33,34]. The person-centred perspective is the view that rehabilitation aims to empower patients by taking self-identified goals as its point of departure and by including the patient as a partner in her/his rehabilitation process. This process is defined as the process after a stroke, where the person is engaged to attain his/her vital life goals and achieve an optimal degree of social well-being and functioning supported by health services and social networks [35]. Furthermore, it includes supporting the discovery and awareness of disabilities, learning problem-solving strategies, addressing and solving everyday problems, planning and performing activities needed to reach goals and reflecting on goal attainment, i.e., self-management [36]. Person-centred care takes place in a context (everyday life) in which the built environment is viewed as important [37,38,39] but is unexplored.

The concept of health builds on the theory of salutogenesis [40], which focuses on resources for health and health-promoting processes rather than on disease with regard to pathogenesis. In salutogenesis, experiences of health and well-being are hypothesized to depend on general resistance resources, which are postulated to be personal factors that also include the assets available in people’s environment. Hence, health can be promoted by creating environments that support people in identifying their internal and external resources and learning how to use and reuse them in order to achieve vital goals in their everyday lives [41].

### 1.3. Preliminary Work

Our study protocol builds on a research project where we study the influence of the built environment on people with stroke at acute stroke units. Moving away from the acute stroke setting, we have completed a pilot study in which we followed 15 patients discharged from hospital to home. In the pilot study, we noted the need to use a valid and reliable instrument to assess housing accessibility. We also learnt that the patients inclusion in rehabilitation planning is vital in terms of receiving good care at home. As scientific literature in this field is lacking, we have expanded our previous research so as to investigate the role of the built environment in patient participation, documentation and healthcare planning (understanding how a person-centred rehabilitation process can be supported).

## 2. Methods 

### 2.1. Study Design

**Figure 1:**
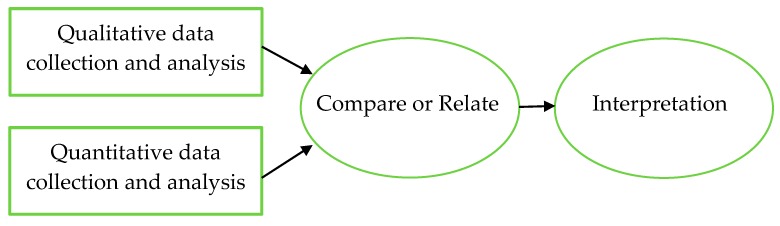
The convergent parallel mixed-methods approach.

The study has an explorative design, and it adopts an ethnographic approach [42]. We will use convergent parallel mixed methods [43] in which qualitative and quantitative data are collected in parallel, analysed separately and then merged to synthesise the contexts, mechanisms and outcomes that are important to support the rehabilitation process in the home (see Figure 1). The study involves collecting data from several sources. For study aims 1–3, data will be collected (questionnaires, semi-structured interviews, observations) with approximately 40 persons during home visits 3 months after stroke onset. This qualitative and quantitative data will help us to understand facilitators/barriers in the built environment, how recovery in terms of engagement in meaningful activities and participation is supported and allow us to measure outcomes such as self-efficacy. Study aim 4 includes two data collections: a medical record documentation analysis and focus group discussions. The documentation analysis will reveal whether and how the built environment has been taken into account during rehabilitation planning and the focus group discussions will provide important knowledge related to how communication between healthcare providers can be improved. For study aim 5, these data will be supplemented by workshops to provide an in-depth understanding of how the built environment for stroke rehabilitation could be designed to support a person-centred rehabilitation process, from the perspectives of people with stroke, those healthcare staff working with them and their significant others.

### 2.2. Participants and Setting

Patients with stroke will be recruited from three healthcare settings. A sample of approximately 40 patients referred to home rehabilitation from the stroke early supported discharge team (ESD) and continued rehabilitations at home will be eligible for inclusion and invited to participate. The patients will be informed about the study by the ESD team before they leave the hospital. Patients who express an interest and willingness to participate will receive an informed consent letter and will be contacted to schedule an interview with the research team. After informed consent has been obtained, the patients will be asked for their permission to contact a significant other, who will then be contacted and invited to participate in the study. Staff at stroke units at the participating hospitals and rehabilitation staff who are responsible for care at home will be eligible for inclusion. Approximately 10 staff members at each site will be included.

The inclusion criteria for the persons with stroke will be that they (1) have had a mild to moderate stroke according to the Barthel Index [44] and were discharged to their homes directly from the stroke unit and (2) are able to communicate and answers questions. Inclusion criteria for the significant others will be that they are (1) identified by persons with stroke as their significant other, (2) willing to participate and (3) able to communicate and answer questions. The inclusion criteria for the staff will be that they have worked in the target organization > 6 months. To explore study aim 4, we will scrutinize the medical records belonging to the 15 people diagnosed with stroke that participated in the pilot study (see Section 1.3).

## 3. Data Collection

**Figure 2:**
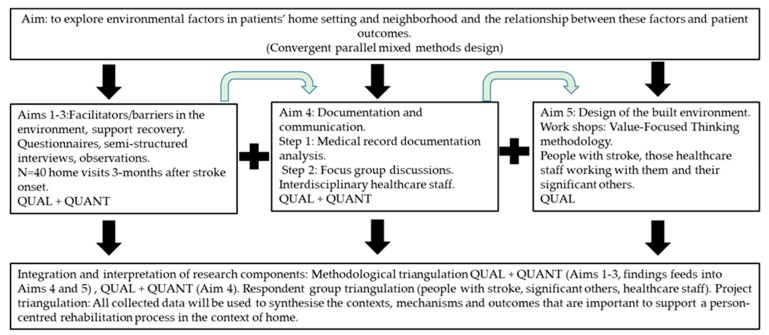
Methods flow chart.

As described in Figure 2, data for study aims 1–3 will be collected by means of self-reported questionnaires, observations and interviews at home visits 3 months after stroke onset. To investigate whether and how the built environment has been taken into account during discharge planning meetings, and to investigate the concurrence between the identified prerequisites to return home and the actual environmental conditions (study aim 4), data will be obtained through patients’ medical records. Building on the results from this part of the project, we will conduct a series of focus group interviews [45] with interdisciplinary staff so that key success factors related to documentation and communication of environmental factors in between care contexts can be revealed.

We will use a value-focused thinking methodology [46] to identify what interdisciplinary staff, persons with stroke and their significant others consider important in the design of the built environment to support a person-centred process from the stroke unit to the home environment (study aim 5). Data will be collected in two workshops, facilitated by authors Kylén, Elf and a PhD student. Before the workshops, the participants will be given a pre-reading document with information about a person-centred stroke rehabilitation process and value-focused thinking. Workshop 1 involves a brainstorming activity in which important factors in the design of the built environment will be revealed. Objectives will be organised in a hierarchy and then synthesised. During workshop 2, the synthesis will be reviewed and discussed, which will result in a framework of what is important in the design of the built environment to support a person-centred rehabilitation process from the stroke unit to the home.

To be able to administrate the Housing Enabler instrument, the researcher who will conduct the data collection (first author MK) have completed a four-day training course and practical training in using the instrument [47].

Study outcomes for the persons with stroke and the staff will be assessed by the following questionnaires and methods, presented below and in Table 1. 

### 3.1. Descriptive Variables

Data on medical information before discharge (including presence of complications, e.g., falls), socio-demographics and functioning will be collected from the medical records. Data will also be collected from the Swedish Stroke register at the point of data collection, 3 months after stroke onset (e.g., perceived mobility and health, depression, types of support or assistance from health services/municipality after stroke, ability to manage activities of daily life).

### 3.2. Health Outcomes

#### 3.2.1. Patients’ Reported Health and Perceived Impact of Stroke

Patients’ reported health status will be measured with the visual analogue scale from the EuroQual 5 (EQ-5D) [48]. The scale rates participants’ perceived overall health from 0 (worst imaginable health) to 100 (best imaginable health). The EQ-5D has been found to be both sensitive to change and valid [49]. Global recovery after stroke will be measured using one item (ranging from 0 no recovery to 100 maximum recovery) from the Stroke Impact Scale (SIS) [50]. The scale has been validated in stroke studies. Perceived problems after stroke will be collected from the Swedish Stroke register and through semi-structured interview questions.

#### 3.2.2. Self-Efficacy

Self-efficacy will be assessed by the General Self-Efficacy scale (GSE), a 10 item self-report scale measuring self-beliefs to cope with a variety of difficult demands in life. Individuals rate their belief in their ability to achieve the 10 items on a 4 point rating scale ranging from 1 (not at all true) to 4 (exactly true), where higher scores indicate a greater sense of general self-efficacy [51]. The scale has been translated into Swedish [52] and shown to be valid [53].

#### 3.2.3. Patient Safety

Patient safety will be assessed based on self-reported occurrence of complications (falls, skin breaks, disorientation, re-admission, and infection) between hospital discharge and the time of the data collection.

### 3.3. Social Environmental Aspects

#### Participation in Social Activities and Perceived Loneliness

Engagement in social activities is an important part of the rehabilitation process, which will be captured through semi-structured interviews. The interview questions are based on previous findings [54] regarding post-stroke social needs, e.g., needs for social life, types of social activities, satisfaction with activities and the places where activities are commonly performed. The Frenchay Activities Index (FAI) [55] will be used to develop a semi-structured interview guide regarding participation in everyday social activities. Loneliness will be measured through the question: “Are you ever bothered by feelings of loneliness?”, with four response categories (“nearly always”, “often”, “seldom” and “almost never”) [56].

### 3.4. Objective and Perceived Environmental Aspects

#### 3.4.1. Exploring Environmental Barriers and Accessibility Problems

Data on environmental barriers and accessibility problems will be collected with the Housing Enabler (HE) instrument, which is based on extensive research [57] and has proven to be reliable and valid [58]. The instrument is based on national standards for housing design and is administrated in three steps. In step 1, functional limitations (12 items, e.g., visual impairment, poor balance, reduced fine motor skill) and dependence on mobility devices (2 items) are dichotomously assessed (present/not-present). Step 1 provides a profile of functional limitations and renders a sum score of functional limitations (range 0–12) and dependence on mobility devices (range 0–2). Collected data in step 1 (12 + 2 items) can also be used as a health variable. Step 2 includes an observation and a detailed dichotomous (present/not-present) assessment of 161 environmental barriers; 87 in the home, 46 at the entrance and 28 in the immediate surrounding environment. Step 2 provides a sum score of environmental barriers (range 0–161/or divided in the 3 subdomains) as well as a detailed description of present environmental barriers. Based on the results from step 1 and 2, step 3 involves a person–environment fit analysis. An instrument specific software calculates a total score that quantifies the magnitude of accessibility problems in a particular case, and forecasts the load triggered by a specific combination of functional limitations and environmental barriers. A higher score indicates more accessibility problems. In cases with no functional limitations/dependence on mobility devices present, the total score is always 0, regardless of environmental barriers. The environmental barriers that cause the greatest accessibility problems can be calculated and ranked. 

#### 3.4.2. Housing Adaptations

Data on housing adaptations will be collected through standardized interview questions. First, the study participants will be asked whether they have had any adaptations made in their home (yes/no). If yes, they will be asked to report the location and whether the adaptation has had any positive or negative influences on activities in daily life (e.g., easier to preform daily activities, more independent/not needing as much help, able to continue living in the present home, the adaptation had insufficient effect, the situation is worse). If applicable, participants will also answer an open-ended interview question reflecting on their participation and shared decision making in the process of receiving the adaptation in their home.

#### 3.4.3. Barriers and/or Facilitators of the Built Environment

Patients’ activity and interactions with the environment and rehabilitation activities will be observed using participants’ observations of real-life situations as well as semi-structured interviews (e.g., Can you tell me about a typical day at home, what do you do?). The observations will be conducted as a complement to the Housing Enabler assessment (see Section 3.4.1).

#### 3.4.4. Place Attachment and Meaning of Home 

Patients´ place attachment will be captured through semi-structured interview questions (e.g., How long have you lived here? Do you feel “home”? What do you like/dislike about living here? Does it, or has it, changed how you feel about yourself?). Meaning of home will be assessed with the 28 item meaning of home questionnaire. The questionnaire captures four aspects: behavioural (6 items), for example “being at home for me means doing everyday tasks”; physical (7 items), for example “being at home for me means feeling that home has become a burden”; cognitive/emotional (10 items), for example “being at home for me means feeling safe”; and social (5 items), for example “being at home for me means being excluded from social and community life”. Each item is rated on a scale ranging from 0 (strongly disagree) to 10 (strongly agree). In the data analysis, some items are reversed. Higher scores indicate a stronger bonding/attachment to home [59]. The 28 item scale has shown an acceptable level of internal consistency, Cronbach’s a = 0.78 [34].

### 3.5. Patient Participation in Decisions on Care and the Role of the Environment

#### 3.5.1. Participation in Rehabilitation Planning and Shared Decisions

Patients’ perceived participation in the decision process regarding their goals, rehabilitation outcomes and their partnership with health professionals will be captured through semi-structured interviews and study specific questions (e.g., “Can you tell me about when you were discharged from hospital?”, “Did you attend a discharge-planning meeting?”, “How did you feel and what did you talk about during the meeting?”).

#### 3.5.2. Documentation of Environmental Factors

Data to investigate whether and how the built environment has been taken into account during discharge planning meetings will be obtained through patients’ medical records. We will scrutinize patient records from the specialized stroke unit and home rehabilitation documentation, as well as the informal communication in-between these care units. Data will be collected regarding environmental factors such as technical aids, potential accessibility problems and need for housing adaptations.

#### 3.5.3. The Experiences of Patients, Significant Others and Staff in Terms of the Rehabilitation Process

The experiences of patients, significant others and staff in terms of the rehabilitation process will be collected through semi-structured interviews and workshops. Open-ended questions such as “How do you feel about ‘training’ at home? What do you usually do?” will be used.

## 4. Analysis

The experiences of the environment and the rehabilitation process from the perspective of the various stakeholders will be analysed by content analysis [60]. To synthesise the work groups’ hierarchies, audio recordings and field notes from workshop 1 will be analysed. In order to refine the framework, researchers in the project will review the hierarchy and definitions that constitutes the framework until consensus is reached [46]. Data from the observations and interviews will be analysed regarding facilitators and/or barriers in the built environment. Presence of facilitators and/or barriers and interactions for the rehabilitation process will be analysed using the ICF as a framework. A deductive content analysis approach [60] will be used to analyse the concurrence between the identified prerequisites to return home and the actual environmental conditions in patients’ medical records. Quantitative data will be analysed by descriptive statistics and the measure of accessibility will be used to explore accessibility problems in depth [57].

### Synthesis

The study will uncover patterns of facilitators and/or barriers in the environment that can generate a model of how the factors are interrelated in order to attain a person-centred rehabilitation process. By synthesising these findings and those regarding the experiences of different built environments from perspectives of several stakeholders, we will provide a more complete picture of which environmental factors are important for a person-centred rehabilitation process. This could supply a broad basis for understanding what will work, for whom and in what context, and it will support the further development and implementation of rehabilitation environments that are flexible and can be tailored to the different needs of patients and significant others.

## 5. Ethics and Dissemination

The study will be conducted in accordance with the Helsinki Declaration and has been approved by the Ethical Board in Uppsala (2015/389). The patient group is potentially vulnerable, and specific considerations must be taken to avoid harm. We will follow ethical requirements according to the Swedish Research Council and Swedish legislation. The data will be archived safely and in accordance with standard rules for data handling. Informed consent will be obtained from all participants and anonymity ensured. This will be reinforced verbally as well as by means of written information at the start of the home visit. The participants will also be informed about confidentiality and their right to withdraw from the study at any time. In order to monitor data quality, meetings will be held on a regular basis with an experienced researcher (last author ME).

We have a number of venues to disseminate our results as we work and are established as researchers in the fields of healthcare, planning and construction and architecture. We will use our position to communicate the results and knowledge obtained during and when the project is completed. For people with stroke and their families, we will use networks of end-user organizations for stroke care to disseminate findings, e.g., in newsletters, websites and present our findings at annual meetings for their members. We will report our findings in national and international journals and conferences to reach healthcare professionals, planners and architects. Policy makers will be reached through publications in opinion papers, national and international journals, invite them to a workshop at the end of the project, and inform them of our findings through personal communication and social media (e.g., Twitter). We easily reach students as we teach in healthcare courses and programs (undergraduate to doctoral level), e.g., nursing, medicine, physiotherapy, occupational therapy and architecture on a regular basis. Thus, the results will reach people who operate in a future healthcare and design context. We will publish the results in scientific journals and at conferences. Selected high-impact and highly cited health and design journals will be targeted. Possible conferences and meetings for the dissemination of the results are the UK Stroke Forum and the European Stroke Organisation Conference, CIB (Construction and Building Research) annual conference. The Forum for Healthcare Architectures organises conferences twice a year. The Forum has a website where news spreads about new research. The Centre for Healthcare Architecture at Chalmers also arranges an international conference, ARCH (Architecture Research Care & Health Conference), every other year. Twitter and other social media will be used for project updates. A newsletter (twice per year) will be used to communicate outcomes and progress to stakeholders and others interested in the project.

## 6. Expertise

The researchers in this project have been selected based on their specific expertise and their proven record of accomplishments. The group members have experience leading and conducting complex multi-stakeholder studies and projects, especially in stroke care. The researchers have also conducted several studies combining methods and perspectives from healthcare and architecture. The researchers’ expertise covers all aspects of the project design (qualitative/quantitative), the theoretical constructs underpinning the study, and research, clinical, and architecture expertise to ensure project deliverables.

## 7. Strengths and Limitations of This Study

The design of our research project permits for the collection of data from multiple sources using a variety of methods, which will contribute to the richness of the data and analysis. We will use collected data to synthesise the contexts, mechanisms and outcomes that are important to support the rehabilitation process in the home. Our team is interdisciplinary with expertise within health science, stroke rehabilitation, architecture, design, environmental psychology and environmental gerontology. The research design contains collaborations with clinicians, persons with stroke and their significant others which will improve knowledge translation to stroke rehabilitation professionals, ultimately optimizing a person-centred care rehabilitation process from the hospital to the home.

As with any study that contains observations, there is potential for bias in terms of the influence the observer might have on the study participants. However, previous research has shown that after a few minutes of being observed, the observed participant returns to their normal behaviours [61]. In this study, we will only include a limited number of patients with stroke, all living in the south of Sweden. A word of caution when it comes to generalizing the results to other Swedish or international groups is therefore needed. In our project, patients with severe complications after stroke will be excluded, which is a limitation. However, the focus is on at home rehabilitation and patients with severe medical complications often receive in-patient care, rather than at home care.

## 8. Significance

Health services in the home are becoming an essential part of the entire care system [62] and the proposed project addresses several concerns and challenges for policy makers of health services for people with chronic conditions, e.g., the need to improve the transition between inpatient and outpatient care, person-centred care and accessibility to care [63,64]. To date, the majority of the research exploring the relationship between the built environment and health outcomes has focused on acute care [18]. Much less research has been conducted within the field of rehabilitation and especially the home environment, even though reports highlight the importance of including the built environment as a means to improve healthcare quality, and thus, to increase person-centred care [27]. In addition, actors in healthcare, architecture and sustainable spatial planning must engage more in the planning and design of the environment for home care. The environment must optimize the effectiveness of care and ensure that the highest quality of care can be provided for patients with frail health and their families. However, the collaboration among healthcare and actors for sustainable buildings is underdeveloped. One way to address this is multidisciplinary research projects that explore the built environment and its interrelationship with the person. Our project will generate empirically grounded knowledge about the impact of environmental factors on the rehabilitation process and association with promoting the health and well-being for a large number of people. In this project, we focus on people with stroke. Stroke make up the third leading cause of hospital care, and as people with stroke represent complex and varying impairments, it is likely that the results can be transferred to other groups of disabilities and may be extended well beyond Sweden. Furthermore, rehabilitation services are increasingly provided in the home environment for most people. In the future, ordinary housing must be able to be adapted to include appropriate care, which should also include good working environments for the health services work force. An increasing ageing population in the society, extensive development of new care models and new technology for care and treatment requires consideration of new care situations for advanced home care and rehabilitation. This will place new demands on the housing market to include flexibility and adaptability to different life situations. In the present project, we will integrate research on architecture, environmental psychology and health services to bridge the knowledge gap, and thus initiate an interdisciplinary approach to studying housing quality, care processes and environments in order to provide new operational knowledge in close collaboration with stakeholders in society. International research cooperation will provide valid strategic support to decision-makers in healthcare, planning and construction. This project brings together Swedish and international experts committed to improving care models and design in this neglected field and will break down silos and build capacity for the future. Thus, the long-term benefits for the research area will be the development of methods for research on the environmental factors that influence health. Providing high-quality science on environmental factors in the home setting poses challenges for research that requires multidisciplinary knowledge. Evidence-based design is a growing research area, and it is essential to explore various methods in order to be able to study the link between health and environmental factors [16].

## 9. Conclusions

Previous evidence suggests that planned discharge tailored to individual needs can reduce adverse events and promote competence in self-management. However, the environmental factors that may play a role in a patient’s recovery process remain unexplored. The present study is exploring environmental factors in patients’ home settings and neighbourhoods and the relationship between these factors and patient outcomes. The first patients are recruited. The study is ongoing.

## Figures and Tables

**Table 1:** Overview of participants, outcomes, methods and questionnaires.

Participants	Outcome	Methods/Questions	Reference
Persons with stroke	Health	EQ-5D	[48]
	Impact of stroke	SIS	[50]
	Self-efficacy	GSE	[51,52]
	Safety	Medical records and interviews	
	Decision-making and goal setting/participation	Interviews	
	Meaning of home	Meaning of home questionnaire	[59]
	Participation in everyday activities	Interview questions inspired by FAI	[55]
	Perceived loneliness	Interview question	[56]
	Environmental barriers and accessibility problems	The Housing Enabler Instrument	[47]
	Housing adaptations	Study-specific questions	
	Activity and interactions with the environment	Observations and interviews	
	Experience of the environment/care and rehabilitation process	Interviews	
	Documentation of environmental factors	Medical records	
Significant others	Experience of the environment/care and rehabilitation process	Workshops	
	Experience of cross-site communication routines	Focus group interviews	
Staff	Experience of the environment/care and rehabilitation process	Workshops	
	Experience of cross-site communication routines	Focus group interviews	

## Abbreviations

CIB	Construction and Building
ESD	Early Supported Discharge
EQ-5D	EuroQual 5
FAI	Frenchay Activities Index
GSE	General Self-Efficacy
HE	Housing Enabler
ICF	International Classification of Functioning, Disability and Health
OECD	The Organisation for Economic Co-operation and Development
SIS	Stroke Impact Scale
WHO	World Health Organization

## References

[1] Organisation for Economic Cooperation and Development (OECD) Health Ministerial Statement. The Next Generation of Health Reforms. http://www.oecd.org/newsroom/oecd-health-ministerial-statement-the-next-generation-of-healthreforms.htm.

[2] Landers S., Madigan E., Leff B., Rosati R.J., McCann B.A., Hornbake R., MacMillan R., Jones K., Bowles K., Dowding D. (2016). The future of home health care. Home Health Care Manag. Pract..

[3] WHO Framework on Integrated People-Centred Health Services. http://www.who.int/servicedeliverysafety/areas/people-centred-care/en/.

[4] SOU Effektiv Vård (Effective Care). www.sou.gov.se/wp-content/uploads/2016/01/SOU-2016_2_Hela4.pdf.

[5] Marcheschi E., Von Koch L., Pessah-Rasmussen H., Elf M. (2018). Home setting after stroke, facilitators and barriers: A systematic literature review. Health Soc. Care Community.

[6] Ringelstein E.B., Chamorro A., Kaste M., Langhorne P., Leys D., Lyrer P., Thijs V., Thomassen L., Toni D. (2013). ESO Stroke Unit Certification Committee. European Stroke Organisation recommendations to establish a stroke unit and stroke center. Stroke.

[7] Wottrich A.W., von Koch L., Tham K. (2007). The meaning of rehabilitation in the home environment after acute stroke from the perspective of a multiprofessional team. Phys. Ther..

[8] Pearson M., Hunt H., Cooper C., Shepperd S., Pawson R., Anderson R. (2015). Providing effective and preferred care closer to home: A realist review of intermediate care. Health Soc. Care Community.

[9] Thrift A.G., Cadilhac D.A., Thayabaranathan T., Howard G., Howard V.J., Rothwell P.M., Donnan G.A. (2017). Global stroke statistics. Int. J. Stroke.

[10] SOU Multipla Hälsoproblem Bland Personer över 60 år: en Systematisk Litteraturöversikt om Förekomst, Konsekvenser och Vård. https://www.riksdagen.se/sv/dokument-lagar/dokument/statens-offentliga-utredningar/multipla-halsoproblem-bland-personer-over-60-ar_GYB348.

[11] Ostwald S.K., Godwin K.M., Ye F., Cron S.G. (2013). Serious adverse events experienced by survivors of stroke in the first year following discharge from inpatient rehabilitation. Rehabil. Nurs..

[12] von Koch L., Holmqvist L.W., Wottrich A.W., Tham K., de Pedro-Cuesta J. (2000). Rehabilitation at home after stroke: A descriptive study of an individualized intervention. Clin. Rehabil..

[13] Ytterberg C., Thorsén A.M., Liljedahl M., Holmqvist L.W., von Koch L. (2010). Changes in perceived health between one and five years after stroke: A randomized controlled trial of early supported discharge with continued rehabilitation at home versus conventional rehabilitation. J. Neurol. Sci..

[14] Singam A., Ytterberg C., Tham K., von Koch L. (2015). Participation in complex and social everyday activities six years after stroke: Predictors for return to pre-stroke level. PLoS ONE.

[15] Nanninga C.S., Meijering L., Schönherr M.C., Postema K., Lettinga A.T. (2015). Place attachment in stroke rehabilitation: A transdisciplinary encounter between cultural geography, environmental psychology and rehabilitation medicine. Disabil. Rehabil..

[16] Elf M., Fröst P., Lindahl G., Wijk H. (2015). Shared decision making in designing new healthcare environments-time to begin improving quality. BMC Health Serv. Res..

[17] Ulrich R., Zimring C., Zhu X., DuBose J., Seo H.B., Choi Y.S., Quan X., Joseph A. (2008). A review of the research literature on evidence-based healthcare design. HERD.

[18] Shepley M., Gerbi R.P., Watson A.E., Imgrund S., Sagha-Zadeh R. (2012). The impact of daylight and views on ICU patients and staff. HERD.

[19] Nordin S., McKee K., Wallinder M., von Koch L., Wijk H., Elf M. (2017). The physical environment, activity and interaction in residential care facilities for older people: A comparative case study. Scand. J. Caring Sci..

[20] Connellan K., Gaardboe M., Riggs D., Due C., Reinschmidt A., Mustillo L. (2013). Stressed spaces: Mental health and architecture. HERD.

[21] Vårdanalys Vården ur Befolkningens Perspektiv-Jämförelser Mellan Sverige och 10 Andra Länder. https://www.vardanalys.se/wp-content/uploads/2017/12/V%C3%A5rden-ur-befolkningens-perspektiv-2016-en-j%C3%A4mf%C3%B6relse-mellan-Sverige-och-tio-andra-l%C3%A4nder.pdf.

[22] Regeringskansliet Patient Law 2014:821. https://www.global-regulation.com/translation/sweden/2987465/patient-law-%25282014%253a821%2529.html.

[23] Cameron J.I., Tsoi C., Marsella A. (2008). Optimizing stroke systems of care by enhancing transitions across care environments. Stroke.

[24] Cameron J.I., Naglie G., Silver F.L., Gignac M.A. (2013). Stroke family caregivers’ support needs change across the care continuum: A qualitative study using the timing it right framework. Disabil. Rehabil..

[25] SBU Patientdelaktighet I Hälso-Och sjukvården. En Sammanställning av Vetenskapliga Utvärderingar av Metoder som kan Påverka Patientens Förutsättningar för Delaktighet. https://www.sbu.se/sv/publikationer/sbu-bereder/patientdelaktighet-i-halso-och-sjukvarden/.

[26] Baker A. (2001). Crossing the quality chasm: A new health system for the 21st century. BMJ.

[27] WHO International Classification of Functioning, Disability and Health (ICF). https://www.who.int/classifications/icf/en/.

[28] Rowles G.D., Bernard M., Rowles G.D., Bernard M. (2013). The meaning of and significance of place in old age. Environmental Gerontology Making Meaningful Places in Old Age.

[29] Korpela K.M., Ylén M., Tyrväinen L., Silvennoinen H. (2009). Stability of self-reported favourite places and place attachment over a 10-month period. J. Environ. Psychol..

[30] Oswald F., Wahl H.W., Rowles G.D., Chaudhury H. (2005). Dimensions of the meaning of home in later life. Home and Identity in Late Life: International Perspectives.

[31] Marcheschi E., Laike T., Brunt D., Hansson L., Johansson M. (2015). Quality of life and place attachment among people with severe mental illness. J. Environ. Psychol..

[32] Kylén M., Löfqvist C., Haak M., Iwarsson S. (2019). Meaning of home and health dynamics among younger older people in Sweden. Eur. J. Ageing.

[33] Haak M., Kylén M., Ekström H., Schmidt S.M., Horstmann V., Elmståhl S., Iwarsson S. (2015). Relationships between perceived aspects of home and symptoms in a cohort aged 67–70. Arch. Gerontol. Geriatr..

[34] Kylén M., Schmidt S.M., Iwarsson S., Haak M., Ekström H. (2017). Perceived home is associated with psychological well-being in a cohort aged 67–70 years. J. Environ. Psychol..

[35] Ekstam L., Uppgard B., Von Koch L., Tham K. (2007). Functioning in everyday life after stroke: A longitudinal study of elderly people receiving rehabilitation at home. Scand. J. Caring Sci..

[36] Vårdanalys Patient-Centredness in Sweden’s Health System-an External Assessment and Six Step for Progress. https://www.vardanalys.se/wp-content/uploads/2017/11/2012-7-Patientcenteredness-v7-0-web.pdf.

[37] Henriksen K., Isaacson S., Sadler B.L., Zimring C.M. (2007). The role of the physical environment in crossing the quality chasm. Jt. Comm. J. Qual. Patient Saf..

[38] Kogan A.C., Wilber K., Mosqueda L. (2016). Person-centered care for older adults with chronic conditions and functional impairment: A systematic literature review. J. Am. Geriatr. Soc..

[39] McCormack B., McCance T.V. (2006). Development of a framework for person-centred nursing. J. Adv. Nurs..

[40] Antonovsky A. (1987). The Jossey-Bass Social and Behavioral Science Series and the Jossey-Bass Health Series. Unraveling the Mystery of Health: How People Manage Stress and Stay Well.

[41] Eriksson M., Lindström B. (2008). A salutogenic interpretation of the Ottawa Charter. Health Promot. Int..

[42] Pink S., Morgan J. (2013). Short term ethnography: Intense routes to knowing. Symb. Interact..

[43] Kettles A.M., Creswell J.W., Zhang W. (2011). Mixed methods research in mental health nursing. J. Psychiatr. Ment Health Nurs..

[44] Govan L., Langhorne P., Weir C.J. (2009). Categorizing stroke prognosis using different stroke scales. Stroke.

[45] Krueger R., Casey M. (2009). Focus Groups: A Practical Guide for Applied Research.

[46] Keeney R.L. (1992). Value-Focused Thinking: A Path to Creative Decisionmaking.

[47] Iwarsson S., Slaug B. (2010). Housing Enabler–A Method for Rating/Screening and Analysing Accessibility Problems in Housing. Manual for the Complete Instrument and Screening Tool.

[48] EQ-5D. http://www.euroqol.org/.

[49] Golicki D., Niewada M., Buczek J., Karlińska A., Kobayashi A., Janssen M.F., Pickard A.S. (2015). Validity of EQ-5D-5L in stroke. Qual. Life Res..

[50] Duncan P.W., Wallace D., Min Lai S., Johnson D.E., Embretson S., Jacobs Laster L. (1999). The stroke impact scale version 2.0. Stroke.

[51] Schwarzer R., Jerusalem M., Weinman J., Wright S., Johnston M. (1995). Generalized self-efficacy scale. Measures in Health Psychology: A User’s Portfolio. Causal and Control Beliefs.

[52] Koskinen-Hagman M., Schwarzer R., Jerusalem M. (1999). Swedish Version of the General Self-Efficacy Scale. http://userpage.fu-berlin.de/*health/swedish.htm.

[53] Löve J., Moore C.D., Hensing G. (2012). Validation of the Swedish translation of the general self-efficacy scale. Qual. Life Res..

[54] Northcott S., Moss B., Harrison K., Hilari K. (2016). A systematic review of the impact of stroke on social support and social networks: Associated factors and patterns of change. Clin. Rehabil..

[55] Piercy M., Carter J., Mant J., Wade D.T. (2000). Inter-rater reliability of the Frenchay activities index in patients with stroke and their carers. Clin. Rehabil..

[56] Dahlberg L., Agahi N., Lennartsson C. (2018). Lonelier than ever? Loneliness of older people over two decades. Arch. Gerontol. Geriatr..

[57] Iwarsson S., Haak M., Slaug B. (2012). Current developments of the Housing Enabler methodology. Br. J. Occup. Ther..

[58] Helle T., Nygren C., Slaug B., Brandt A., Pikkarainen A., Hansen A., Pétursdórttir E., Iwarsson S. (2010). The Nordic Housing Enabler: Inter-rater reliability in cross-nordic occupational therapy practice. Scand. J. Occup. Ther..

[59] Oswald F., Schilling O., Wahl H.W., Fänge A., Sixsmith J., Iwarsson S. (2006). Homeward bound: Introducing a four-domain model of perceived housing in very old age. J. Environ. Psychol..

[60] Hsieh H.F., Shannon S.E. (2005). Three approaches to qualitative content analysis. Qual. Health Res..

[61] McDonald S. (2005). Studying actions in context: A qualitative shadowing method for organizational research. Qual. Res..

[62] Regeringskansliet God och Nära Vård-En Primärvårdsreform. https://www.regeringen.se/rattsliga-dokument/statens-offentliga-utredningar/2018/06/sou-201839/.

[63] Vårdanalys VIP i Vården?–Om Utmaningar i Vården av Personer Med Kronisk Sjukdom. https://www.vardanalys.se/wp-content/uploads/2017/12/2014-2-VIP-i-v%C3%A5rden.pdf.

[64] Vårdanalys Vården ur Patienternas Perspektiv. https://www.vardanalys.se/rapporter/varden-ur-patienternas-perspektiv/.

